# A Polyclonal Selex Aptamer Library Directly Allows Specific Labelling of the Human Gut Bacterium *Blautia producta* without Isolating Individual Aptamers

**DOI:** 10.3390/molecules27175693

**Published:** 2022-09-03

**Authors:** Hu Xing, Yiting Zhang, Markus Krämer, Ann-Kathrin Kissmann, Marius Henkel, Tanja Weil, Uwe Knippschild, Frank Rosenau

**Affiliations:** 1 Institute of Pharmaceutical Biotechnology, Ulm University, Albert-Einstein-Allee 11, 89081 Ulm, Germany; 2 Max-Planck-Institute for Polymer Research Mainz, Ackermannweg 10, 55128 Mainz, Germany; 3 Cellular Agriculture, TUM School of Life Sciences, Technical University of Munich, Gregor-Mendel-Straße 4, 85354 Freising, Germany; 4 Department of General and Visceral Surgery, Surgery Center, Ulm University, Albert-Einstein-Allee 23, 89081 Ulm, Germany

**Keywords:** aptamer, *B. producta*, biosensor

## Abstract

Recent studies have demonstrated that changes in the abundance of the intestinal bacterium *Blautia producta*, a potential probiotic, are closely associated with the development of various diseases such as obesity, diabetes, some neurodegenerative diseases, and certain cancers. However, there is still a lack of an effective method to detect the abundance of *B. producta* in the gut rapidly. Especially, DNA aptamers are now widely used as biometric components for medical testing due to their unique characteristics, including high chemical stability, low production cost, ease of chemical modification, low immunogenicity, and fast reproducibility. We successfully obtained a high-affinity nucleic acid aptamer library (B.p-R14) after 14 SELEX rounds, which efficiently discriminates *B. producta* in different analysis techniques including fluorometric suspension assays or fluorescence microscopy from other major gut bacteria in complex mixtures and even in human stool samples. These preliminary findings will be the basis towards aptamer-based biosensing applications for the fast and reliable monitoring of *B. producta* in the human gut microbiome.

## 1. Introduction

As a complex ecosystem, the gut microbiome influences many aspects of host physiology, such as disease development, drug metabolism and regulation of the immune system [1]. Among these microbiota *Blautia* is one of the most abundant genera of intestinal bacteria [2,3,4], especially *B. producta*, which is a strictly anaerobic, inactive, and 1.0–1.5 μm × 1.0–3.0 μm spherical or oval-shaped intestinal bacterium. It has both heterotrophic and autotrophic properties as it can use CO, H_2_/CO_2_, and carbohydrates as energy sources [5,6]. It has received wider attention in recent years due to its prominent contribution in alleviating inflammatory and metabolic diseases and its antimicrobial activity against specific microorganisms [7,8]. According to the data provided by Kakiyama et al., it was confirmed that the reduction of *Blautia* abundance in the intestine might lead to the development of cirrhosis [9]. Using 16S rRNA gene-based next generation sequence analysis to determine the overall structure of the microbiota in colorectal cancer patients and healthy controls, reduction of *Blautia* numbers in the gut was found to be associated with the risk of colorectal cancer [10]. Moreover, Mabrok et al. found that *B. producta* promotes the conversion of secoisolariciresinol diglucoside (SDG) to enterodiol (ED) and enterolactone (EL) by colonizing the gut of germ-free mice with specific bacteria [11]. These two active substances have been shown to reduce the risk of breast cancer in women significantly [12,13,14]. A 454-pyrosequencing analysis of microbial communities in fresh stool and mucosal samples from patients with Crohn’s disease, ulcerative colitis, and healthy subjects suggested that changes in *Blautia* abundance in the gut might increase the risk of inflammatory bowel disease [15]. More recently, an investigation of the relationship between visceral fat and BMI (body mass index) and gut microbes revealed a significant inverse relationship between *Blautia* abundance in the gut and visceral fat area (VFA), which argues that a decrease in *Blautia* abundance may contribute to the development of obesity [16]. In addition, based on numerous previous studies, it has been demonstrated that *B. producta* secretes lantibiotics to inhibit the growth of vancomycin resistant *Enterococci* (VRE), the main pathogenic bacteria that cause hospital-acquired infections. The most noteworthy of these is the narrow antimicrobial spectrum of lantibiotics that can specifically target VRE without affecting other commensal bacteria [17,18,19]. In recent studies, the safety of *B. producta* was further evaluated in terms of virulence genes, acute oral toxicity, etc., and it was found that *B. producta* could be a promising probiotic for the prevention of various diseases such as obesity, infections, and some cancers [20].

Oligonucleotide aptamers as potent novel recognition elements have now made their way into application in the field of (bio)sensing due to their high stability, ease of chemical modification, design and synthesis, and their efficient binding constants compared to antibodies [21,22,23]. Nucleic acid aptamers with high affinity to specific target molecules are usually isolated from synthetic random aptamer libraries using an in vitro evolution technique called SELEX (Systematic evolution of ligands by exponential enrichment) [22,23,24]. With their unique shape-forming property, these single-stranded oligonucleotides adopt complex 3D structures that allow for high affinity to their dedicated targets and enable the highly specific interactions with them [24]. Since its first application by Tuerk et al. and Ellington et al. in 1990, the SELEX technique has evolved over the years into different forms, thus making the screening process more efficient [24,25]. Among the cell-specific aptamer selection methods, the most commonly used method using whole cells as targets is called the Cell-SELEX. The first Cell-SELEX was used for African trypanosomes [26], where researchers screened for high-affinity aptamers from a library of RNA aptamers that bind to the flagellar pouch of the bloodstream stage of African trypanosomes. Currently, Cell-SELEX has been applied to various types of tumor cells [27,28], virus-infected cells [29], human cells [30], and parasites [31] for specific aptamer screening. It is a cyclic and iterative process involving binding, elution, and amplification steps. The SELEX process of DNA aptamers starts with a random library of 10^12^–10^16^ single-stranded DNA (ssDNA) molecules, each sequence containing a random region and two fixed regions for binding the primers during amplification. The random library is incubated with the target molecule for DNA aptamer screening, unbound sequences are removed, DNA/target cell complexes are isolated, PCR amplifies the corresponding DNA sequences, and the next round of screening is performed. The candidate aptamers are then tested after multiple rounds of selection and evaluated for target affinity [23].

**Figure 1:**
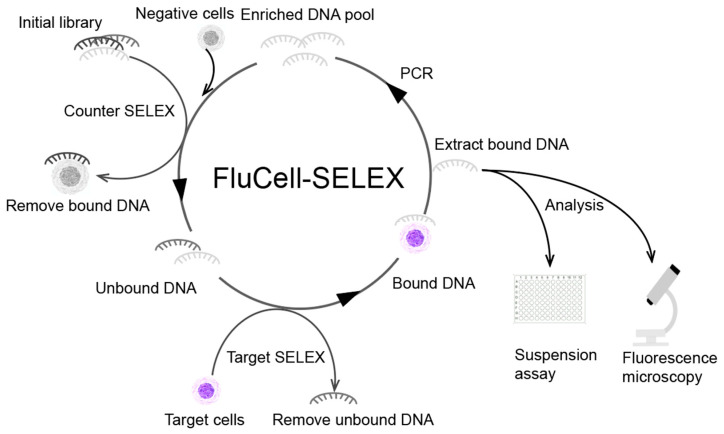
The initial library was first incubated in a counter SELEX with a mixture of bacteria including *A. muciniphila*, *A. steroricanis*, *B. producta*, *R. microfusus*, and *R. intestinalis*. Aptamers attached to negative or non-target cells were removed by centrifugation. Through this process, the selection pressure was increased to improve the screening efficiency. Subsequently, the unbound aptamers were further incubated with *B. producta* by Target-SELEX to obtain aptamer sub-populations. Aptamers not attached to *B. producta* were removed by simple washing, and then the aptamer sub-populations were amplified by PCR after elution to obtain a new focused DNA pool. This screening process was repeated to obtain a polyclonal library that specifically binds the target cells, which was finally analyzed by suspension assays and fluorescence microscopy.

We have recently developed the so-called FluCell-SELEX using Cyanine 5 (Cy5) fluorescently labelled aptamers, and the binding of aptamers to target cells can be characterized by changes in fluorescence intensity throughout the screening process [23]. Here, we describe the use of FluCell-SELEX to evolve aptamer libraries that specifically can recognize and label *B. producta* (Figure 1). The use of “focused” or “polyclonal” libraries represents a significant simplification of the evolution of specific binding moieties for several biological assays as well as for the design of electronic biosensors. The elimination of all sequencing and analysis efforts to obtain individual aptamers represents a further advantage of using already focused libraries with respect to the possible speed of technological development of functional and specific binding units in the case of emerging health-threats such as novel pathogens. The resulting library was found to allow discrimination of *B. producta* as the dedicated target cells from other prominent gut bacteria and to retrace amounts of *B. producta* in mixtures of these bacteria including *Akkermannsia muciniphila*, *Allobaculum stercoricanis*, *Parabacteroides distasonis*, *Rikenella microfusus* and *Roseburia intestinalis*. To our knowledge, we are the first to develop a specific aptamer library to identify this auspicious probiotic bacterium of probably essential importance for human health. This specific aptamer library as a potent entity of robust affinity molecules also will open the way for the future development of easily accessible and cost-effective next generations of electronic or optical biosensors for the detection of *B. producta* in clinical diagnostics.

## 2. Results

**Figure 2:**
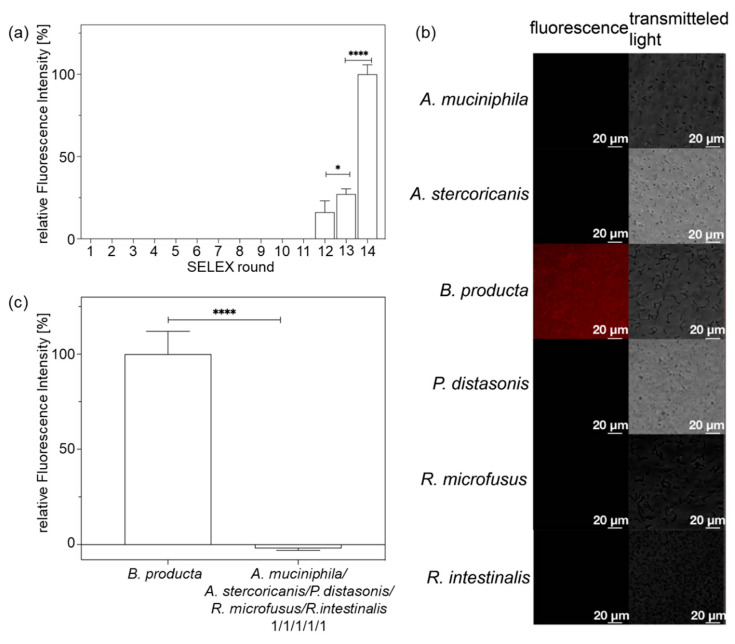
Specificity of the aptamer libraries. (**a**) Monitoring of the evolution process. After each round of selection, equal amounts of aptamer libraries were co-incubated with *B. producta* (1 mL, OD_600_ = 1), and then the Cy5-labelled aptamers were freed from the surface of *B. producta* by heating after three washes with 1 × DPBS. The progressively enhanced fluorescence signal showed a gradual enrichment of the aptamer library recognizing *B. producta*. *p* values < 0.05 were considered significant. * denotes *p* < 0.01, and **** *p* < 0.0001. (**b**) Fluorescence microscopy of Cy5-labelled polyclonal aptamer library B.p-R14 bound to *B. producta*. The binding of polyclonal aptamer library B.p-R14 to *B. producta* showed a strong fluorescent signal, whereas the other five intestinal bacteria, *P. distasonis*, *A. muciniphila*, *R. microfusus*, *A. stercoricanis*, and *R. intestinalis* as controls showed weak fluorescent signals. (**c**) Specificity analysis of the aptamer library B.p-R14. The control was a mixture of the other five intestinal bacteria *P. distasonis*, *A. muciniphila*, *R. microfusus*, *A. stercoricanis*, and *R. intestinalis*, according to 1:1:1:1:1. The aptamer library B.p-R14 showed high specificity in identifying *B. producta* compared to the control. *p* values < 0.05 were considered significant. **** denotes *p* < 0.0001. All experiments were conducted as triplicates (*n* = 3).

The FluCell-SELEX cycle was run for 14 rounds with increasing stringency by multiplying washing steps after aptamer binding, increasing BSA and tRNA concentrations for blocking unspecific binding, decreasing the aptamer concentration, and applying counter selection steps with mixtures “contaminating” control bacteria according to the scheme described in Table 1. The development of the binding specificity was monitored in each round by measuring the fluorescence that could be eluted from *B. producta* cells after incubation with the respective aptamer libraries. The relative fluorescence intensity provided by the libraries of each round by binding to the cells and thus representing the ability of the aptamer ensemble comprising these libraries to recognize *B. producta* was markedly enhanced after the 14 rounds of screening (Figure 2).

**Figure 3:**
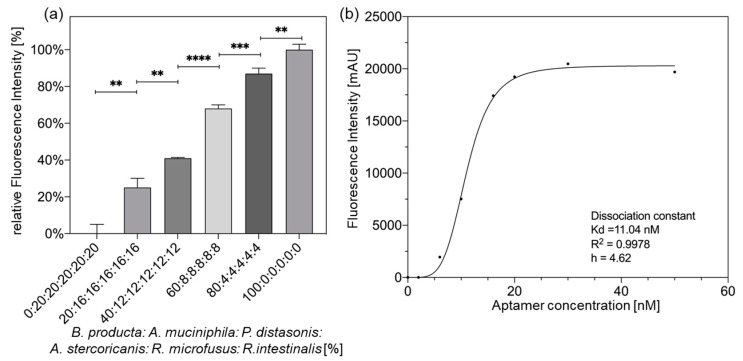
Specific detection of *B. producta* using the polyclonal aptamer library B.p-R14. (**a**) Detection of *B. producta* by Cy5-labeled polyclonal aptamer library B.p-R14 in a mixture of intestinal bacteria including *A. muciniphila*, *P. distasonis*, *A. stercoricanis*, *R. microfusus*, and *R. intestinalis*, all mixed at the same optical density but different ratios. *p* values < 0.05 were considered significant. ** indicates *p* < 0.01, *** *p* < 0.001 and **** *p* < 0.0001. All experiments were conducted as triplicates (*n* = 3). (**b**) One site-specific binding of a polyclonal aptamer library B.p-R14. *B. producta* was incubated with different concentrations of the aptamer library B.p-R14. The resulting curves were fitted by non-linear regression using the one site-specific binding model in GraphPad Prism 8. The polyclonal aptamer library B.p-R14 dissociation constant was calculated to be 11.04 nM, the Hill coefficient was 4.62, and the deviation of the coefficient of determination was 0.9978.

The fluorescence intensity during the repetitive SELEX rounds obviously first increased in the background, then reaching the detection threshold of the assay and becoming detectable from round 12 gaining significant differences of fluorescence between rounds 12 and 13. Compared with round 13 of selection, the relative fluorescence intensity clearly increased (nearly 4-fold) in round 14, indicating that a rapid and effective enrichment was achieved by significantly increasing the selection pressure in the evolution of *B. producta* specific aptamer populations within the libraries (Figure 2a). In addition, the aptamer library B.p-R14 also showed its ability to precisely label and identify *B. producta* compared to other bacteria *P. distasonis*, *A. muciniphila*, *R. microfusus*, *A. stercoricanis*, and *R. intestinalis* in the intestine. The control showed almost no detectable fluorescent signal (Figure 2b,c). Thus, based on these properties, it was possible to use the aptamer library B.p-R14 to track *B. producta* in different proportions of mixed bacteria precisely. For the aptamer library B.p-R14, the intensity of the fluorescence signal detected in the experiment showed a clear linear relationship with the amount of *B. producta* in the mixture. Therefore, the amount of *B. producta* in the bacterial mixture could be judged based on the intensity of the fluorescence signal detected in the practical application (Figure 3a). Equal amounts of *B. producta* were co-incubated with different concentrations of the aptamer B.p-R14 and the detected fluorescence intensity was fitted to the concentration of the aptamer B.p-R14 with the typical one-site-specific binding using the Hill equation. The dissociation constant of the aptamer B.p-R14 was found to be 11.04 nM, and the Hill coefficient was 4.62, which indicates that it has a high affinity for *B. producta* and conforms to the typical cooperative binding mode [32]. Therefore, once aptamers bind to *B. producta*, its affinity to other aptamers of the library increases. Meanwhile, the deviation of the coefficient was observed to be 0.9978, which further indicates the reliability of the experimental data (Figure 3b). In summary, the polyclonal aptamer library B.p-R14 has the potential to label and detect *B. producta* sensitively and accurately.

**Figure 4:**
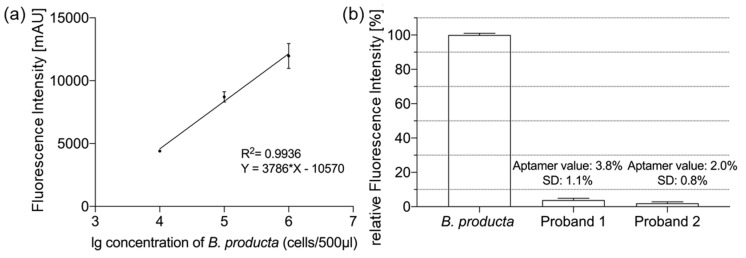
(**a**) Aptamer B.p-R14 sensitivity assay, the decrease of fluorescence intensity depends on the decrease of cell concentration, the solid line is the calibration curve obtained from the correlation between cell concentration and fluorescence intensity change, the bacterial concentration is positively correlated with fluorescence intensity when the number of bacteria in 500 µL 1x DPBS varies in the range of 10^4^ to 10^6^ cells. The detection limit of the aptamer library B.p-R14 for *B. producta* was 10^4^ cells. (**b**) *Blautia* abundance in male and female fecal samples based on the aptamer library B.p-R14 and 16S rDNA NGS. The actual value represents the actual *Blautia* content in fecal bacteria as determined by NGS. The target value shows the *Blautia* abundance in fecal bacteria as determined by the aptamer library B.p-R14. The SD stands for the standard deviation during the measurement of the target value. All experiments were conducted as triplicates (*n* = 3).

To further quantify the sensitivity of the aptamer library B.p-R14, we varied the bacterial concentration and generated a calibration curve (Figure 4a), which showed a positive correlation between bacterial concentration and fluorescence intensity (R^2^ = 0.9936) when the number of bacteria in the 500 µL reaction system was varied in the range of 10^4^ to 10^6^ cells. This implies that the fluorescence intensity increased with increasing bacterial concentration at this time. We determined that the detection limit of the aptamer library B.p-R14 for *B. producta* in this 500 µL volume was 10^4^ cells, which demonstrated the sensitivity of the aptamer library for identifying *B. producta* and suggested the possibility to quantify relevant amounts of *B. producta* in fecal samples. To further test this ability of the aptamer library B.p-R14 to detect the abundance of *B. producta* in fecal bacteria, we first obtained the exact amount of *Blautia* in the feces of two healthy volunteers (proband 1 and proband 2; Tables S3 and S4) by 16S rDNA Next Generation Sequencing (NGS). Subsequently, we used 5 pmol of the aptamer library B.p-R14 in a 500 µL 1 × DPBS with adjusted numbers of *B. producta* (10^8^ cells) as a reference and fecal bacteria from the stool samples (10^8^ cells), respectively, to perform the binding ability assay. The NGS results showed that the actual amount of *Blautia* in the fecal bacteria of healthy male and female volunteers was 2.79% and 2.32%. Compared to 3.8% and 2.0% based on the analysis of the highly specific aptamer library B.p-R14, this showed similar results (Figure 4b). Considering the fluorescence assay and the possible bias in the experimental procedure, we have reason to believe that the aptamer library B.p-R14 is feasible and reliable for the detection of *Blautia* abundance in feces in practice. The assay takes only 30 min and requires only 10^8^ cells of fecal bacteria, which opens up new possibilities for rapid micro detection of fecal bacteria.

## 3. Discussion

Since changes in the abundance of *B. producta* in the intestine are closely associated with the development of several diseases, the rapid determination of its content in the intestinal microbiota has become an urgent problem. However, traditional detection techniques are long and complicated and cannot meet the requirements of rapid microbial detection. Therefore, developing and designing of a novel, rapid microbial detection method using aptamer-based biosensors have become a popular endeavor [33].

To the best of our knowledge, we are the first to develop a polyclonal aptamer library that specifically labels and detects *B. producta,* after 14 rounds of Cell-SELEX, in complex mixtures as well as in human stool samples. While exhibiting a low dissociation constant (K_d_ = 11.04 nM), strong and high affinity binding was ensured by the evolved polyclonal library B.p-R14. Taking the dynamic nature of aptamers into account, an equilibrium exists between the unfolded state and the binding competent state, where the binding competent state is responsible for high affinity binding of the target with characteristic low K_d_ values [32]. The evolved polyclonal library B.p-R14 efficiently labels its dedicated target *B. producta* in fluorometric microtiter plate assays and in fluorescence microscopy when alone as well as in defined mixtures with five other major gut microorganisms and even in highly complex human stool samples. Compared to identification of the *Blautia* content in human samples by sequencing of 16S rRNA genes, the relative numbers determined by the calibrated fluorometric aptamer analysis differed only marginally with obtained NGS values of 2.79% and 2.32% and aptamer-based relative *B. producta* amounts of 3.8% and 2.0% for proband 1 and proband 2, respectively. However, gaining fast and reliable species- or genus-level classification is difficult using NGS sequencing, as 16S rRNA genes do not have a single hypervariable region which can discriminate between all species [34]. Without the determination of the binding specificity of B.p-R14 towards other *Blautia* species, it hence can be suggested that aptamer binding occurs mainly below the genus level.

The reliable and rapid detection of dysbiosis in the human microbiome in clinical microbiology can be seen as a prototypical application of aptamers in the future, which is possible not only because of their high specificity and affinity, but also because of their optimization potential for different assay systems [35]. As a probiotic with great potential, *B. producta* plays a pivotal role in preventing and treating many diseases. The specific binding of polyclonal aptamer libraries to target cells on the surface of individual cells depends on specific components of the outer surface of the cell membrane, such as lipopolysaccharides and outer membrane proteins [36]. The polyclonal aptamer library B.p-R14 possibly offers high labeling flexibility as well as assay fidelity, especially when compared to a single aptamer. The signal fluctuation caused by a single aptamer when identifying highly complex targets like whole cells, can be compensated by polyclonal libraries, thus significantly improving the reliability of detection results [23]. We recently showed that a wider sequence space of a polyclonal library enables a higher precision for target recognition and increases the performance in biosensor technologies by three orders of magnitude compared to single aptamers [37]. Therefore, polyclonal aptamer libraries are expected to be a new strategy with many applications in medical diagnostics and biosensor technologies [38]. We believe that its potential superiority could pave the way for introducing polyclonal aptamer libraries as the next generation of potent molecular binding ligands and we envision the development of new complex biosensors for clinical diagnostics.

## 4. Materials and Methods

### 4.1. Cell Lines and Cell Culture

All bacteria strains *A. muciniphila* mucT (DSM-22959), *A. stercoricanis* (DSM-13633), *R. intestinalis* (DSM-14610), *B. producta* (DSM-29491), *P. distasonis* (DSM-29491), and *R. microfusus* (DSM-15922) were cultivated under anaerobic conditions in Schaedler Broth medium at 37 °C.

### 4.2. Aptamer Library and Primers

The random ssDNA library (TriLink BioTechnologies, Inc, San Diego, California, USA) and primers (Eurofins Genomics, Ebersberg, Germany) were designed and synthesized (Table 1) with a primer complementary binding sequence containing 23 bases at both the 5′- and 3′-ends and a 40-base random sequence in between (N_(40)_). The forward primer Cy5-P1 for Cy5 fluorescent labelling at the 5′-end is identical to the fixed sequence at the 5′-end of the ssDNA library, and the reverse primer Biotin-P_2_ for Biotin labelling is complementary to the fixed sequence at the 3′-end of the ssDNA library.

### 4.3. Identification of the Initial Library

The random ssDNA library and primers were identified by 2% agarose gel electrophoresis to determine whether the library and primers were successfully constructed. The positions of the double-stranded DNA bands were also determined as the reference standard for the target bands of the PCR products in subsequent experiments.

### 4.4. Cell SELEX

Cell-SELEX included counter-SELEX as well as target-SELEX. For counter-SELEX, the negative bacteria used here was the bacteria mixture including *A. muciniphila* mucT, *A. steroricanis*, *P. distasonis*, *R. intestinalis*, and *R. microfusus*. Non-binders to negative bacteria could be subsequently screened by target-SELEX to obtain an aptamer library specifically targeting *B. producta*. The traditional Cell-SELEX could be divided into the following steps.

#### 4.4.1. Cell Pretreatment

The overnight incubated bacteria were centrifuged at 7500× *g* for 1 min, subsequently washed three times with 1x DPBS buffer, and finally adjusted to the OD_600_ of the bacterial solution of 1.

#### 4.4.2. Activation of Aptamer Library

The aptamer library was added to 500 µL 1x DPBS, subsequently incubated at 95 °C for 5 min, in an ice bath for 5 min, and then at room temperature for 20 min to facilitate correct folding of the aptamers. The native 3D structure of the aptamers can be ensured after complete denaturation and direct refolding.

#### 4.4.3. Screening

The activated aptamer library was incubated with negative cells for 1 h at 37 °C and then centrifuged at 3000× *g* for 2 min. The aptamer library in the supernatant was further incubated with *B. producta* at 37 °C. BSA (100 mg/mL) and tRNA (10 mg/mL) with increasing amounts over the selection process were added to reduce non-specific binding. Subsequently, the supernatant was removed through centrifugation at 3000× *g* for 2 min. Finally, the pellet was washed with 1x DPBS to discard unbound aptamers (see Supplementary Table S1).

#### 4.4.4. Elution

The pellet (from 4.4.3) was resuspended in 100 µL1x DPBS and by incubating at 95 °C for 5 min, the 3D structure of aptamers was disrupted to separate aptamers from the cells. The aptamers bound to *B. producta* were finally collected by centrifugation at 11,000× *g* for 2 min.

#### 4.4.5. Acquisition of Secondary Libraries

PCR amplified the collected aptamers (from Section 4.4.4) and the amplification conditions were as follows (see Supplementary Table S2). The PCR product was subsequently incubated overnight with the streptavidin labelled magnetic beads (Qiagen, Hilden, Germany). The ssDNA was isolated in 0.1 M NaOH, 0.1 M NaH_2_PO_4_ and 1 × DPBS after one washing step with 1 × DPBS.

#### 4.4.6. Binding Assay

*B. producta* was preprocessed by the above method (see Section 4.4.1). 5 pmol of Cy5-labeled aptamer library was added in 500 µL of 1x DPBS. After activation (see Section 4.4.2), the aptamer library was incubated with *B. producta* (1 mL OD_600_ = 1) for 30 min at 37 °C. Subsequently, the supernatant was removed by centrifugation at 3000× *g* for 2 min, and the pellet was post-processed by the earlier described method (see Section 4.4.4). Finally, the fluorescence intensity was measured using an Infinite F200 spectrophotometer (TECAN, Männedorf, Switzerland) to determine the screening status (excitation: 637 nm and emission: 670 nm).

### 4.5. Determination of High Specificity Aptamer Libraries

#### 4.5.1. Specificity Analysis

All bacterial solutions, including *A. muciniphila* mucT, *A. steroricanis*, *P. distasonis, B. producta*, *R. intestinalis*, and *R. microfusus* were preprocessed by the above method (see Section 4.4.1) and 5 pmol of Cy5-labeled aptamer library was added in 500 µL of 1 × DPBS. After activation (see Section 4.4.2), the aptamer library was incubated with *B. producta* (1 mL OD_600_ = 1) for 30 min at 37 °C. Subsequently, the supernatant of each group was removed by centrifugation at 3000 rpm for 2 min, and the pellet was post-processed as described above (see Section 4.4.4). By comparing the fluorescence intensity of each group with PBS as the negative control group, the specificity of the aptamer library was analyzed.

#### 4.5.2. Affinity Analysis

The bacterial solution of *B. producta* was preprocessed by the above method (see Section 4.4.1). The fluorescence intensity of the aptamer library was determined by incubating *B. producta* (1 mL OD_600_ = 1) with varying concentrations of activated aptamers in 500 µL of 1 × DPBS (see Section 4.4.2) for 30 min at 37 °C. By fitting the fluorescence intensity dependence on the aptamers’ concentration using Graphpad Prism 8 (GraphPad Software, CA, USA), the dissociation constant (Kd) was finally determined.

#### 4.5.3. Fluorescence Microscopy

All bacterial solutions, including *A. muciniphila* mucT, *A. steroricanis*, *B. producta*, *P. distasonis*, *R. intestinalis*, and *R. microfusus* were pre-treated by the above method (see Section 4.4.1). 25 pmol of the aptamer library was added in 500 µL of 1x DPBS. After activation, as described earlier (see Section 4.4.2), the library was incubated with each microorganism (1 mL OD_600_ = 1) for 30 min at 37 °C. The supernatant was subsequently removed after centrifugation at 3000× *g* for 2 min. The pellet was washed once with 500 µL of 1x DPBS and then resuspended in 500 µL of 1x DPBS. Afterward, 100 µL of each bacterial solution was transferred to a 96-well microplate. Finally, the fluorescence imaging of each cell was gained using fluorescence microscopy, which was performed using a Leica DMi8 coded (Leica Microsystems CMS GmbH, Wetzlar, Germany) at ×20 magnitude under transmitted light with the Y5 filter.

### 4.6. Analysis of B. producta Abundance in Fecal Samples

#### 4.6.1. Human Samples

Fecal samples from two healthy lean probands were used in this study. Volunteers were recruited from Ulm University and signed a written informed consent form. Permission was received from the local ethics committee of Ulm University (no. 30/20). In addition, the study was designed and conducted following the regulations governing the use of human study participants and in strict accordance with the standards set by the Declaration of Helsinki.

#### 4.6.2. Stool Bacteria Extraction

Stool samples were weighed, added to extraction buffer (1x PBS) and vortexed for 1 min until no fecal pellet was visible. The fecal pellets were then removed by filtration, and the filtrate was centrifuged at 6000× *g* for 1 min, followed by washing three times with 1x PBS buffer and finally adjusted to the OD_600_ of the bacterial solution of 1.

#### 4.6.3. Next Generation Sequencing (NGS)

Fecal samples were collected using the INTEST PRO kit (Biomes Laboratory, Wildau, Germany) and then measured and analyzed by Biomes Laboratory using 16S rRNA NGS for different fecal bacterial abundance (Supplementary Tables S3 and S4).

#### 4.6.4. Analysis of *B. producta* Abundance in Fecal Samples Based on a Highly Specific Aptamer Library

*R. intestinalis* (see Section 4.4.1) and stool samples (see Section 4.6.2) should be pre-treated. The fluorescence intensity was determined by incubating *B. producta* (10^8^ cells) and fecal bacteria (10^8^ cells) individually with 5 pmol activated aptamers in 500 µL of 1 × DPBS (see Section 4.4.2) for 30 min at 37 °C.

## 5. Conclusions

In summary, we provided the first polyclonal aptamer library with specificity towards the human gut bacterium *B. producta* and we describe the possibility to use this library in different microbiological analyses. We showed the specific aptamers maintained their binding functionality towards the target even in human stool samples. Aptamers evolved in SELEX processes, and in particular the direct use of polyclonal libraries without extensive selection of individual sequences, may represent an important opportunity to develop potent binding moieties for various diagnostic tools for the characterization of microbiome dysbiosis in the future including a new generation of electronic biosensors. Such evolution of specific polyclonal libraries is extremely fast and thus this technology may equip the health sciences with valuable opportunities to react quickly also to sudden threats such as the occurrence of pandemic situations, by accelerating the development of specific assays for the respective pathogens. Consequently, based on the *B. producta* library presented here, our next goal is to develop novel aptamer-based (bio)sensors as new clinical diagnostic tools for the rapid detection of gut bacterial abundance.

## Figures and Tables

**Table 1:** DNA sequences of the random DNA library with 40-base random sequence, forward and reverse primer sequences in 5′-3′ orientation.

**Name**	**Sequence 5′-3′**
Random ssDNA library	TAGGGAAGAGAAGGACATATGAT-N_(40)_-TTGACTAGTACATGACCACTTGA
Cy5-P_1_	Cy5-TAGGGAAGAGAAGGACATATGAT
Biotin-P_2_	Biotin-TCAAGTGGTCATGTACTAGTCAA

## Data Availability

Not applicable.

## Supplementary Materials

The following are available online at https://www.mdpi.com/article/10.3390/molecules27175693/s1, Table S1: Conditions of all SELEX rounds of *B. producta*, including the amount of aptamer library, counter SELEX, target SELEX, incubation conditions, washing times, and the amount of BSA/Trna; Table S2: (**a**) PCR protocol for cell-SELEX, in which the reaction components are assembled as described below. The reagents in the following order: PCR water, Herculase II-buffer, dNTPs, template DNA, primers, and Herculase were added in the PCR tube to the given amount. The final volume should be 50 µL. Meanwhile, a negative control reaction without template DNA was prepared to ensure the accuracy of the experiment. (**b**) PCR steps for cell-SELEX. A single PCR run will undergo 25 cycles. The first step is the denaturation step, in which the double-stranded DNA template molecule is made single-stranded with a temperature of 94 °C. The second step is an annealing step with a temperature of 56 °C, in which the primers bind to complementary sequences in the single-stranded DNA. The final elongation step with the temperature of 72 °C is when the DNA polymerase adds bases complementary to the template to the bound primers. The newly DNA strand is extended to the end of the template area; Table S3: Next Generation Sequencing results proband 1; Table S4: Next Generation Sequencing results proband 2.
