# Supplementary material for: A Polyclonal Selex Aptamer Library Directly Allows Specific Labelling of the Human Gut Bacterium Blautia producta without Isolating Individual Aptamers
Source: Molecules. 2022 Sep 3;27(17):5693. doi: 10.3390/molecules27175693 (PMC9458011; doi:10.3390/molecules27175693)
Supplement: Supplementary file 1 [file molecules-27-05693-s001.zip › molecules-1883355-supplementary.pdf]

**Supplementary Materials:**

**Table S1**. Conditions of all SELEX rounds of *B. producta*, including the amount of aptamer library, counter SELEX, target SELEX, incubation conditions, washing times, and the amount of BSA/tRNA.

| SELEX rounds | Aptamer(pmol) | Counter SELEX (Incubation conditions) | Target SELEX (Incubation conditions) | Wash times | BSA/tRNA (pmol) |
|---|---|---|---|---|---|
| 1 | 500 | - | 250μL $OD_{600}$ = 1 (37 °C for 1h) | 1 | 600 |
| 2 | 10 | - | 250μL $OD_{600}$ = 1 (37 °C for 1h) | 1 | 900 |
| 3 | 10 | - | 250μL $OD_{600}$ = 1 (37 °C for 1h) | 2 | 1200 |
| 4 | 10 | - | 250μL $OD_{600}$ = 1 (37 °C for 1h) | 2 | 1500 |
| 5 | 10 | - | 250μL $OD_{600}$ = 1 (37 °C for 1h) | 3 | 1800 |
| 6 | 10 | 250 μL $OD_{600}$ = 2 *R. microfusus* (37 °C for 1h) | 250μL $OD_{600}$ = 1 (37 °C for 1h) | 4 | 2100 |
| 7 | 10 | 50 μL $OD_{600}$ = 2 each bacterium (37 °C for 1h) | 250μL $OD_{600}$ = 1 (37 °C for 1h) | 6 | 2400 |
| 8 | 10 | 50 μL $OD_{600}$ = 2 each bacterium (37 °C for 1h) | 250μL $OD_{600}$ = 1 (37 °C for 1h) | 6 | 2700 |
| 9 | 10 | 50 μL $OD_{600}$ = 2 each bacterium (37 °C for 1h) | 250μL $OD_{600}$ = 1 (37 °C for 1h) | 6 | 3300 |
| 10 | 10 | 50 μL $OD_{600}$ = 2 each bacterium (37 °C for 1h) | 250μL $OD_{600}$ = 1 (37 °C for 1h) | 6 | 3600 |
| 11 | 10 | 50 μL $OD_{600}$ = 2 each bacterium (37 °C for 1h) | 250μL $OD_{600}$ = 1 (37 °C for 1h) | 6 | 3900 |
| 12 | 5 | 50 μL $OD_{600}$ = 2 each bacterium (37 °C for 1h) | 250μL $OD_{600}$ = 1 (37 °C for 1h) | 6 | 4200 |
| 13 | 5 | 50 μL $OD_{600}$ = 2 each bacterium (37 °C for 1h) | 250μL $OD_{600}$ = 1 (37 °C for 0.5 h) | 6 | 4500 |
| 14 | 5 | 500 μL $OD_{600}$ = 2 each bacterium (37 °C for 1h) | 250μL $OD_{600}$ = 1 (37 °C for 0.5 h) | 6 | 4500 |

1. The BSA (100 mg/Lm) and tRNA (10 mg/mL) were incubated with targeted cells as a competitor, which can avoid non-specific binding to the cell surface. 2. Counter-selection: Aptamer library was incubated with *A. muciniphila* mucT, *A. steroricanis*, *R. intestinalis*, *P. distasonis* and *R. microfusus*. 3. Target SELEX: Aptamer library was incubated with *B. producta*.

**Table S2:** (**a**) PCR protocol for cell-SELEX, in which the reaction components are assembled as described below. The reagents in the following order: PCR water, Herculase II-buffer, dNTPs, template DNA, primers, and Herculase were added in the PCR tube to the given amount. The final volume should be 50 µL. Meanwhile, a negative control reaction without template DNA was prepared to ensure the accuracy of the experiment. (**b**) PCR steps for cell-SELEX. A single PCR run will undergo 25 cycles. The first step is the denaturation step, in which the double-stranded DNA template molecule is made single-stranded with a temperature of 94 °C. The second step is an annealing step with a temperature of 56 °C, in which the primers bind to complementary sequences in the single-stranded DNA. The final elongation step with the temperature of 72 °C is when the DNA polymerase adds bases complementary to the template to the bound primers. The newly DNA strand is extended to the end of the template area.

(**a**)

| Component | Volume [µL] | Final concentration |
|---|---|---|
| Herculase II-buffer | 10 | 1x |
| 10 mM dNTP mix | 1.25 | 250 µM |
| Herculase (2 U/µL) | 0.25 | 0.5 U/ 50 µL |
| 100 µM Primer Forward | 0.125 | 0.25 µM |
| 100 µM Primer Reverse | 0.125 | 0.25 µM |
| Template DNA | 1 µL | - |
| PCR $H_20$ | 37.25 µL | - |
| Total | 50.00 µL | - |

(**b**)

| Step | Temp[°C] | Time | iterations |
|---|---|---|---|
| Initial Denaturation | 80.0 | 2 min | - |
| Initial Denaturation | 85.0 | 2 min | - |
| Initial Denaturation | 90.0 | 2 min | - |
| Initial Denaturation | 94.0 | 3 min | - |
| Denaturation | 94.0 | 30 sec | 25 cycles |
| Annealing | 56.0 | 30 sec | - |
| Elongation | 72.0 | 10 sec | - |
| Final-Elongation | 72.0 | 2 min | - |

**Table S3**. Next Generation Sequencing results proband 1.

| Kingdom | Phylum | Class | Order | Family | Genus | Species | Absolute_counts | Relative_counts |
|---|---|---|---|---|---|---|---|---|
| Bacteria | Firmicutes | Clostridia | Lachnospirales | Lachnospiraceae | Blautia | bacterium UC5.1-1D4 | 2142 | 4,66503E+15 |
| Bacteria | Firmicutes | Clostridia | Lachnospirales | Lachnospiraceae | Blautia | obeum | 405 | 8,82044E+15 |
| Bacteria | Firmicutes | Clostridia | Lachnospirales | Lachnospiraceae | Blautia | phocaeensis | 2 | 4,35577E+15 |
| Bacteria | Firmicutes | Clostridia | Lachnospirales | Lachnospiraceae | Blautia | stercoris | 57 | 1,24139E+16 |
| Bacteria | Firmicutes | Clostridia | Lachnospirales | Lachnospiraceae | Blautia | unspecific_Blautia | 1,25098E+16 | 2,72449E+15 |

**Table S4**. Next Generation Sequencing results proband 2.

| Kingdom | Phylum | Class | Order | Family | Genus | Species | Absolute_counts | Relative_counts |
|---|---|---|---|---|---|---|---|---|
| Bacteria | Firmicutes | Clostridia | Lachnospirales | Lachnospiraceae | Blautia | WCE2007 | 123 | 1,44547E+15 |
| Bacteria | Firmicutes | Clostridia | Lachnospirales | Lachnospiraceae | Blautia | bacterium UC5.1-1D4 | 196 | 2,30335E+15 |
| Bacteria | Firmicutes | Clostridia | Lachnospirales | Lachnospiraceae | Blautia | obeum | 832 | 9,77748E+14 |
| Bacteria | Firmicutes | Clostridia | Lachnospirales | Lachnospiraceae | Blautia | phocaeensis | 167 | 1,96255E+15 |
| Bacteria | Firmicutes | Clostridia | Lachnospirales | Lachnospiraceae | Blautia | stercoris | 41 | 4,81823E+15 |
| Bacteria | Firmicutes | Clostridia | Lachnospirales | Lachnospiraceae | Blautia | unspecific_Blautia | 1,95724E+16 | 2,3001E+15 |
